# Real-world data on discordance between estrogen, progesterone, and HER2 receptor expression on diagnostic tumor biopsy versus tumor resection material

**DOI:** 10.1007/s10549-019-05141-y

**Published:** 2019-02-13

**Authors:** A. M. Sofie Berghuis, Carolien H. M. van Deurzen, Hendrik Koffijberg, Leon W. M. M. Terstappen, Stefan Sleijfer, Maarten J. IJzerman

**Affiliations:** 1 Department of Health Technology and Services Research, Faculty of Behavioural, Management and Social Sciences, Technical Medical Centre, University of Twente, Enschede, The Netherlands; 2 Department of Pathology, Erasmus MC Cancer Institute, Rotterdam, The Netherlands; 3 Department of Medical Cell BioPhysics, Faculty of Science and Technology, TechMed Centre, University of Twente, Enschede, The Netherlands; 4 Department of Medical Oncology, Erasmus MC-University Medical Center, Erasmus MC Cancer Institute, Rotterdam, The Netherlands; 5 Cancer Health Services Research Unit, School of Population and Global Health, Faculty of Medicine, Dentistry and Health Sciences, University of Melbourne, Melbourne, Australia

**Keywords:** Discordance, Breast cancer, Estrogen, Progesterone, Human epidermal growth factor receptor 2, Diagnostics

## Abstract

**Purpose:**

The estrogen (ER), progesterone (PR), and HER2 status are essential in guiding treatment decisions in breast cancer patients. In daily life, the ER/PR/HER2 status is expected to be commonly tested twice, i.e., at diagnosis using material from tumor needle biopsies, and after tumor resection using full tumor tissue material. This study explored the discordance of ER/PR/HER2 between tumor needle biopsies and full tumor resection material using real-world patient-level data from Dutch breast cancer patients.

**Methods:**

Pathology reports of 11,054 breast cancer patients were derived from PALGA (Dutch Pathology Registry). Discordance was calculated for multiple combinations of the ER/PR/HER2 receptor status. The influence of patient and tumor characteristics on the probability of having discordant test results was analyzed using multiple logistic regression models (separately for ER, PR and HER2).

**Results:**

For 1279 patients (14.4%), at least one of the receptors (ER/PR/HER2) was determined on both biopsy and tumor tissue material. The majority had concordant test results for ER (*n* = 916; 94.8%), PR (*n* = 1170; 86.7%), and HER2 (*n* = 881; 98.1%). Patients having an ER- and HER2-positive but PR-negative biopsy classification, BR grade III, and < 10% tumor tissue remaining after neoadjuvant therapy (NAT) have the highest probability of ER discordant test results (OR 4.991; *p* = 83.31%). The probability of discordance in PR is based on different sets of patient and tumor characteristics. Potential cost savings from omitting multiple tests if concordance can be perfectly predicted can be up to €205,000 yearly.

**Conclusions:**

Double testing of ER/PR/HER2 is less common than expected. Discordance in ER/PR/HER2 test results between tumor needle biopsy taken at the time of diagnosis and tumor resection material is very low, especially in patients not receiving any form of neoadjuvant therapy. These results imply that a substantial number of tests can potentially be omitted in specific subgroups of breast cancer patients.

## Background

Breast cancer is the most frequently diagnosed form of cancer among women with yearly incidence rates of almost 15,000 women in the Netherlands [1]. Of all women diagnosed with breast cancer, 90% presents with primary breast cancer without distant metastases [2]. In patients with primary breast cancer, the risk of relapse is determined based on factors such as tumor size, tumor grade, and lymph node involvement, and if considered high, patients are candidates for peri-operative systemic treatment aiming to reduce the relapse risk. The type of systemic treatment prescribed is currently mainly dependent on the determination of the ER, PR and HER2 status of the tumor [3]. The ER/PR/HER2 status can be determined on tumor needle biopsy material or on tumor resection specimen using immunohistochemistry (IHC). For the HER2 status, additional in situ hybridization (ISH) test is recommended to confirm the HER2 status in case IHC results are equivocal [4, 5].

In daily life, the ER/PR/HER2 status is determined multiple times in many breast cancer patients: first on specimen derived from a needle biopsy taken for initial diagnosis, and second on the whole tumor specimen obtained by tumor resection [4]. Usually, the receptor status results of the tumor needle biopsy and the tumor resection are expected to be concordant. In patients not receiving any form of neoadjuvant therapy (NAT) between taking the needle biopsy and resection, small series indeed strongly suggest that the concordance between both measurements (biopsy and resection) is relatively high (range 90.8–97.5%) [6, 7]. However, discordance in test results might arise since tumor characteristics can change over time, in particular in patients treated with NAT, or because of sampling or analytical errors [8–10].

Given the importance of assessing the ER/PR/HER2 status for treatment decision making, it is essential to get more insight into the factors that may underlie discordant test results. Apart from consequences for treatment decision making, this is also important to improve the cost-effectiveness of the diagnostic pathway. For those patients in whom it is highly unlikely that the second test would yield a discordant test result or a change in clinical management, one of the ER/PR/HER2 determinations can potentially be omitted. Although the costs of the ER/PR/HER2 tests are relatively low, around €100 for an IHC and between €300 and 400 for an ISH [11, 12], the cumulative costs of the use of such tests can still be high given the large number of patients yearly diagnosed with breast cancer.

Several studies have reported on the discordance in ER/PR/HER2 between tumor needle biopsies and tumor resection. However, most (recent) studies reported on relatively small sample sizes in total and small subgroups of breast cancer patients, or included only patients diagnosed at a single hospital [7, 13–15]. In the study presented here, we have evaluated the discordance in ER/PR/HER2 between tumor needle biopsy and tumor resection material in the majority of invasive breast cancer patients diagnosed in 2016 and 2017 in the Netherlands. In addition, the influences of several tumor and patient characteristics on the probability of discordance in either of the receptors were addressed. Furthermore, potential cost savings due to eliminating over testing in patients with concordant test results were estimated.

## Methods

### Data sources and description

Data on the ER/PR/HER2 status of invasive breast cancer patients were requested from the Dutch Pathology Registry (PALGA), which archives all pathology reports [16]. Since 2009, reporting modules are available for creating those pathological reports. Within these reports, information on patient, tumor, and test characteristics is captured in numerous variables instead of in free text fields, which improves the possibility of analyzing high numbers of reports simultaneously.

Pathology reports of invasive breast cancer patients diagnosed between January 12, 2016 and January 1, 2018 were extracted from PALGA, as on January 12, 2016 a new synoptic reporting module for breast cancer biopsies became available that enabled saving more data in a standardized way.

Performing more than two tests, thereby creating additional excerpt records, can have multiple underlying reasons which are not always well documented within the pathology reports. Therefore, patients having more than two excerpt records were removed from further analysis. Discordance, by definition, can only exist between multiple measurements. Consequently, patients for which only one excerpt record was available were also removed from further analysis.

### Discordance in test results

For each excerpt for which the ER and PR status were tested, the percentage of tumor cells that stained positive for ER and PR and the final classification of the excerpt (positive or negative) were registered. According to the Dutch breast cancer guideline [4], excerpts with 10% or more cells stained positive for ER or PR are classified as being positive. ER or PR classifications that deviate from this protocol, i.e., excerpts that had less than 10% of cells staining positive for ER or PR that were classified as being positive were reclassified according to this 10% threshold.

Tumor needle biopsies will be referred to as biopsies in this study, whereas material derived from surgically removed tumor tissue will be referred to as the tumor resections. Test results between the biopsy and tumor resection were considered discordant, when the final corrected classification per marker, i.e., positive or negative, was different between both excerpts. Discordance was calculated for ER, PR, and HER2 separately, and for ER and PR combined.

### Logistic regression analyses

To estimate whether discordance was more likely to occur in particular subgroups of patients, three independent logistic regression analyses were performed for ER, PR, and HER2. These analyses were performed using the glm function from the stats package (version 3.5.1) in R (version 3.5.1). Discordance in either of the receptors was categorized as a binomial variable and was assigned to be the dependent variable, whereas the type of NAT (i.e., hormonal–chemotherapy, or no neoadjuvant therapy), response to therapy, tumor subtype, Bloom Richardson (BR) grade, TNM stage, ER biopsy classification, percentage of cells positive for ER on biopsy material, PR biopsy classification, percentage of cells positive for PR on biopsy material, HER2 biopsy classification, HER2 IHC result on biopsy material, and HER2 ISH result on biopsy material were assigned as independent variables in the initial model.

In each of the three analyses, a subset of the data was created which contained records of those patients for whom the particular receptor under investigation was tested twice. The logistic regression models were evaluated using the Akaike Information Criterion (AIC) in a stepwise algorithm in which both backward and forward selection of variables was combined. The stepwise approach was performed using the step function from the stats package (version 3.5.1) in R (version 3.5.1) and was started with a null model, in which no variables were manually added to the initial model. The approach was continued with additional steps in which variables were either added forwards or eliminated backwards in each step. The model that was derived when either eliminating or adding a particular variable to the model did not result in a lower AIC of the model was considered to be the model with the best performance.

Before running the logistic regression analyses and to overcome implications of too small patient subgroups, several variables were grouped to maintain a sufficient number of patients in each subgroup. Tumor subtypes were merged into three main categories: ductal carcinoma, lobular carcinoma, and other tumor subtypes. For this reason, also the type of neoadjuvant therapy was reclassified into 4 categories: chemotherapy, hormonal therapy, other or combined therapy, and no therapy. The primary tumor (T), regional lymph nodes (N), and distant metastases (M) stage (TNM) was defined according to the eight edition of the TNM staging system [17]. In this staging system, a N1mi classification can only exist in combination with T1. The N-stage of patients for whom a higher T-stage was recorded in combination with this N1mi was assumed to be a N1-N-stage.

### Potential cost savings of omitting multiple tests

Potential cost savings from avoiding unnecessary tests (i.e., with outcomes similar to the initial test) were calculated. Based on the total number of concordant test results in either ER/PR/HER2, or combinations of those, the number of tests that potentially could have been omitted was calculated. Costs for each test, or combinations of tests, were derived from the Dutch Healthcare Authority (NZa). The ER and PR status are generally tested by using an IHC. In the calculation of the potential cost savings, it was assumed that the costs for testing both ER and PR on the same excerpt were equal to those of a single test for either ER or PR. If HER2 was additionally tested, additional costs of the HER2 antibody were added to the costs of performing an IHC, whereas IHC in combination with either ER, PR, or both was assumed to be equally expensive to performing an IHC for HER2 singularly.

## Results

Patient-level data derived from PALGA included 11,054 patients with invasive breast cancer whom were diagnosed (i.e., had a biopsy) after January 12, 2016. Patients whom had either one excerpt record (*n* = 92; 0.66%) or more than two excerpt records (*n* = 2081; 15.00%) were excluded from further analysis. The final dataset used for further analyses consisted of 8,881 unique patients, whom all had one biopsy and one tumor resection record. An overview of all patient characteristics is provided in Table 1.

**Table 1:** Summarized overview of clinically relevant patient characteristics

Category	Sub-categories	Total number of patients	Therapy			
			No NAT	Chemotherapy	Hormonal therapy	Other therapy
Bloom Richardson grade	Unknown	618 (6.96%)	112 (18.12%)	437 (70.71%)	47 (7.61%)	22 (3.56%)
	Grade I	2464 (27.74%)	2324 (94.32%)	105 (4.26%)	34 (1.38%)	1 (0.04%)
	Grade II	4086 (46.01%)	3827 (93.66%)	205 (5.02%)	49 (1.2%)	5 (0.12%)
	Grade III	1713 (19.29%)	1595 (93.11%)	105 (6.13%)	11 (0.64%)	2 (0.12%)
Response to therapy	No response	7984 (89.9%)	7858 (98.42%)	86 (1.08%)	35 (0.44%)	5 (0.06%)
	< 10% tumor remaining	232 (2.61%)	0 (0.00%)	216 (93.10%)	6 (2.59%)	10 (4.31%)
	10–50% tumor remaining	325 (3.66%)	0 (0.00%)	281 (86.46%)	36 (11.08%)	8 (2.46%)
	> 50% tumor remaining	340 (3.83%)	0 (0.00%)	269 (79.12%)	64 (18.82%)	7 (2.06%)
TNM stage	0	506 (5.70%)	476 (94.07%)	25 (4.94%)	4 (0.79%)	1 (0.20%)
	IA	688 (7.75%)	673 (97.82%)	13 (1.89%)	2 (0.29%)	0 (0.00%)
	IB	4226 (47.58%)	3862 (91.39%)	317 (7.50%)	37 (0.88%)	10 (0.24%)
	IIA	330 (3.72%)	299 (90.61%)	26 (7.88%)	3 (0.91%)	2 (0.61%)
	IIB	1967 (22.15%)	1680 (85.41%)	229 (11.64%)	50 (2.54%)	8 (0.41%)
	IIIA	739 (8.32%)	589 (79.70%)	119 (16.10%)	27 (3.65%)	4 (0.54%)
	IIIB	264 (2.97%)	158 (59.85%)	89 (33.71%)	15 (5.68%)	2 (0.76%)
	IIIC	63 (0.71%)	43 (68.25%)	15 (23.81%)	2 (3.17%)	3 (4.76%)
Tumor type	Ductal	98 (1.10%)	78 (79.59%)	19 (19.39%)	1 (1.02%)	0 (0.00%)
	Lobular	6999 (78.81%)	6166 (88.10%)	715 (10.22%)	91 (1.30%)	27 (0.39%)
	Other	1212 (13.65%)	1067 (88.04%)	101 (8.33%)	41 (3.38%)	3 (0.25%)
	Total	8881 (100%)	7858 (88.48%)	852 (9.59%)	141 (1.59%)	30 (0.34%)

### Discordance

To calculate discordance, only those patients were selected for whom the ER, PR, or HER2 was determined on both biopsy and tumor resection material. For 1279 patients, either ER, PR, or HER2 was determined on both excerpts (14.40%). For those patients who only had 1 ER, PR, or HER2 determination, the majority of patients had their status determined on resection material (ER *n* = 4343; 57.13%, PR *n* = 4363; 57.39%, HER2 *n* = 4772; 62.78%). Discordance in each of the receptors for patients with ER, PR, and HER2 determined on both excerpts is shown in Table 2.

**Table 2:** Discordance in test results between biopsy and tumor resection material

Receptor	Population size^a^	Discordant	Biopsy positive, resection negative	Biopsy negative, resection positive
ER	684 (7.70%)	31 (4.53%)	20 (64.52%)	11 (35.48%)
PR	890 (10.02%)	135 (15.17%)	67 (49.63%)	68 (50.37%)
HER2	707 (7.96%)	6 (0.85%)	0 (0.00%)	6 (100%)

^a^The population size described shows the number of patients for whom each of the specific receptors was determined on both biopsy and tumor resection material

In a substantial number of patients (*n* = 590; 47.20%), the ER and PR receptors were both tested on biopsy and tumor resection material. Discordance in both receptors was found in a small number of patients (*n* = 7; 1.19%).

A substantial percentage of all patients for whom either the ER, PR, or HER2 status was determined on both biopsy and tumor resection material received no NAT (*n* = 1070; 85.60%), which is slightly lower than the relative number of patients not receiving any NAT in the full dataset (*n* = 7858; 88.48%). The percentage of patients having discordance in ER or PR test results is higher in patients receiving either neoadjuvant chemotherapy or hormonal therapy, as shown in Table 3.

**Table 3:** Discordance in ER, PR or HER2 receptor status and neoadjuvant therapy

	Chemotherapy		Hormonal therapy		No NAT	
	Population	Discordant	Population	Discordant	Population	Discordant
ER	96 (11.27%)	9 (9.38%)	17 (12.06%)	1 (5.88%)	568 (7.23%)	21 (3.70%)
PR	110 (12.91%)	21 (19.09%)	30 (21.28%)	12 (40.00%)	745 (9.48%)	99 (13.29%)
HER2	51 (5.99%)	3 (5.88%)	9 (6.38%)	0 (0.00%)	645 (8.21%)	3 (0.47%)

^a^Row sums of the subpopulations that were tested multiple times for each receptor do not add up to population size as presented in column 2, Table 2. Some patients (range 2–5 for the different receptors) received another therapy than chemotherapy or hormonal therapy. None of these patients had discordant test results for any of the receptors

Of those patients with discordance in both the ER and PR receptors (*n* = 7; 1.19%), 1 patient received chemotherapy, whereas all others received no neoadjuvant therapy.

### Influence of tumor and patient characteristics on discordance

Logistic regression models for ER, PR, and HER2 discordance separately were established using the stepwise algorithm. For HER2 discordance, however, the model could not be established properly, as only a few patients had discordance in this receptor (*n* = 6), and was therefore not further evaluated. In the remaining regression analyses (ER and PR), the TNM stages were merged into stage 0–1, stage 2, and stage 3. Patients with different types of NAT, i.e., other than chemotherapy, hormonal therapy, or no NAT, were excluded from this analysis (*n* = 30; 7.67%).

In the regression analysis modeling ER discordance, the stepwise algorithm indicated that including ER and PR biopsy classification, the BR grade, the response to therapy (if any), and the HER2 IHC result of the biopsy results in a model with the lowest AIC. For PR discordance, the best performing model contains the type of neoadjuvant therapy (if any), the ER and PR biopsy classification, the TNM stage, and the tumor type. The derived odds ratios from both models, 95% confidence intervals (CIs), standard errors, and the corresponding p values are shown in Table 4.

**Table 4:** Results of the logistic regression analyses per receptor

	ER discordance	Odds ratio	Std. error	*P* value	CI 2.5%	CI 97.5%
	Intercept^a^	0.050	2.382	0.001	0.008	0.254
ER biopsy classification (ref. biopsy negative, *n* = 463)	Positive (*n* = 463)	3.629	1.654	0.010	1.378	10.073
PR biopsy classification (ref. biopsy negative, *n* = 267)	Positive (*n* = 267)	0.132	1.709	0.000	0.043	0.361
BR grade (ref. unknown BR grade, *n* = 39)	Grade 1 (*n* = 121)	0.990	2.262	0.990	0.199	5.202
	Grade 2 (*n* = 265)	0.191	2.412	0.060	0.031	1.074
	Grade 3 (*n* = 198)	1.482	2.100	0.596	0.367	7.006
ISH biopsy result (ref. no ISH performed, *n* = 420)	Amplified (*n* = 31)	1.399	2.013	0.631	0.293	4.939
	Not Amplified (*n* = 172)	0.191	2.228	0.039	0.028	0.736
NAT (ref. no NAT, *n* = 518)	Chemotherapy (*n* = 90)	3.355	1.695	0.022	1.141	9.245
	Hormonal therapy (*n* = 15)	3.230	3.171	0.310	0.157	22.473

	PR discordance	Odds ratio	Std. error	P value	CI 2.5%	CI 97.5%
	Intercept	0.036	1.874	0.000	0.009	0.113
ER biopsy classification (ref. biopsy negative, *n* = 142)	Positive (*n* = 673)	6.031	1.589	0.000	2.609	16.505
TNM Stadium (ref. stadium 0–1, *n* = 458)	TNM stadium 2 (*n* = 312)	1.910	1.250	0.004	1.236	2.965
	TNM stadium 3 (*n* = 45)	0.973	1.622	0.955	0.353	2.396
NAT (ref. no NAT, *n* = 683)	Chemotherapy (*n* = 104)	2.188	1.361	0.011	1.177	3.959
	Hormonal therapy (*n* = 28)	4.088	1.531	0.001	1.741	9.381
PR biopsy classification (ref. biopsy negative, *n* = 340)	Positive (*n* = 475)	0.500	1.261	0.003	0.317	0.790
IHC biopsy result (ref. no IHC performed, *n* = 131)	IHC 2+ (*n* = 101)	0.927	1.430	0.833	0.457	1.865
	IHC 0 (*n* = 212)	0.479	1.390	0.025	0.250	0.913
	IHC 1+ (*n* = 323)	0.560	1.349	0.053	0.313	1.015
	IHC 3+ (*n* = 48)	0.356	1.833	0.089	0.094	1.065
Tumor type^b^ (ref. different type, *n* = 73)	Ductal (*n* = 618)	1.237	1.585	0.644	0.537	3.370
	Lobular (*n* = 124)	2.146	1.645	0.125	0.855	6.192

^a^The intercept reflects the probability of discordance for the total reference category of patients. For the prediction of ER discordance, these patients have an ER-negative but PR-positive biopsy classification, an unknown BR grade, had no ISH performed on the biopsy material, and did not receive any form of NAT

^b^Both tumor types appear to have a non-significant influence compared to a different type of tumor. This is due to recoding this variable. Ductal and lobular are significantly different from each other

The outcomes of the logistic regression analyses, as shown in Table 4, show that the probability of discordance in the ER receptor is higher for patients with an ER-positive biopsy classification than for patients with an ER-negative biopsy classification (OR 3.629), whereas the probability of discordance in this ER receptor is lower for patients with a PR-positive biopsy classification compared to patients with a PR-negative biopsy classification (OR 0.132). The probability of discordance in the PR receptor is higher for patients with an ER-positive biopsy classification compared to patients with an ER-negative biopsy classification (OR 6.031), whereas the probability of discordance in the PR receptor is lower for patients with a positive biopsy classification for HER2 derived by IHC compared to patients for whom no IHC was performed (OR 0.356).

When combining the different patient and tumor characteristics, specific subgroups of patients can be determined with the highest and lowest probabilities of deriving discordant test results in either of the receptors. Patients with a BR Grade 3, a positive classification of ER on biopsy, and a positive classification of HER2 on biopsy derived by an amplified ISH who did receive neoadjuvant chemotherapy have the highest probability of an ER discordant test result (OR 1.274; *p* 56.01%), whereas patients with a BR Grade 2, a positive classification of PR on biopsy, and a negative classification of HER2 on biopsy derived by a not amplified ISH who did not receive any NAT at all have the lowest probability of an ER discordant test result (OR ≤ 0.005; *p* = 0.02%). Patients with a lobular tumor type staged as TNM stage of 2A or 2B, a positive classification of ER, a negative classification of PR, and no HER2 IHC performed on biopsy and who did receive neoadjuvant hormonal therapy have the highest probability of a PR discordant test result (OR 3.627; *p* = 78.39%), whereas patients with another tumor type than lobular or ductal, a TNM stage 3A, 3B, or 3C, a positive classification of PR on biopsy, and a positive classification of HER2 derived by a 3 + IHC result and who did not receive any form of NAT had the lowest probability of a PR discordant test result (OR 0.006; *p* = 0.62%).

### Potential cost savings

For the majority of patients in whom multiple tests were performed, the test results were concordant, ranging from 84.83% in PR status to a concordance of 99.15% in the HER2 status. The cost of determining the ER and PR status together is €151, whereas additionally determining the HER2 on an IHC is €100 more expensive. The cost of an ISH is approximately €408. Determining the ER, PR, and complete HER2 status (IHC and ISH) results in a cost of approximately €659.

Assuming (hypothetically) that test results can be perfectly predicted for patients with concordant test results, either one of the tests performed can potentially be omitted. In Table 2, the total number of patients having discordant test results was presented. However, as tests can be performed simultaneously, it was additionally calculated how many patients had concordant test results in all receptor statuses (ER, PR, and HER2). A total of 325 full ER/PR/HER2 tests can potentially be omitted, as all receptors were found to be concordant (approximately €214,000). In addition, also ER and PR are often tested simultaneously. A total of 179 tests can additionally be potentially omitted, as 179 patients had a combined ER/PR test and concordant test results for both receptors while not having had two HER2 determinations (approximately €27,000). The number of patients with double tests for ER, PR, or HER2 singularly (double tests in only 1 receptor) was then calculated, resulting in additional cost savings of approximately €169,000. In total, potential cost savings can sum up to approximately €410,000 when concordance in test results can be perfectly predicted, and second tests can therefore safely be omitted. Determining the ER/PR/HER2 status on both biopsy and tumor resection material is currently already selectively performed (*n* = 1279; 14.40%). However, cost savings for this particular patient group (from 2016 till 2018) may still result in yearly cost savings of approximately €205,000. Overall, these results indicate that an average cost saving of up to €320 per patient can potentially be reached.

## Discussion

The majority of patients had two excerpt records in their pathology reports. These results are in accordance with what was expected for most breast cancer patients and in line with the Dutch breast cancer guideline, as usually a biopsy is done to confirm breast cancer after which the tumor is resected in invasive breast cancer. In general, for most patients (*n* = 7631; 85.9%) the ER, PR, and HER2 status were, however, only tested once on either biopsy or tumor resection material. Discordance in the ER/PR/HER2 receptor was shown to be limited, with percentages of patients with discordant test results in either one of the receptors ranging from 0.9 to 15.2%. When testing for both the ER and PR receptors, discordance in test results was also found to be very low (1.19%). The percentages of patients with discordant test results were substantially higher in patients who received any form of NAT (i.e., chemotherapy or hormonal therapy) compared to those patients who did not receive any form of NAT.

Two separate multivariable stepwise logistic regression analyses were performed to calculate the probability of deriving discordant test results for the ER and PR receptors as a function of patient and tumor characteristics that are available directly after the biopsy. Even though the numbers of patients having discordant test results in specific subgroups are relatively low, the data suggest that several of these variables significantly influence the probability of deriving a discordant test result. However, before predicting discordance using such models, these should be validated, which is often complex or even impossible in datasets in which a minority of observations is used to predict the outcome. In this study, small percentages of patients had a discordant test result, especially for ER and even more for HER2, whereas this percentage was slightly higher for PR. Consequently, validating the model for PR discordance may be easier, given the larger number of expected discordant test results. Although potentially feasible and valid in predicting discordance, singly testing the PR receptor is clinically less relevant than single tests for the ER and PR receptors. Therefore, such a model would not serve its initial clinical aim, which is of course predicting discordance in clinically meaningful receptors. However, the regression analyses do have provided valuable insight into the combination of patient and tumor characteristics which appear to be influencing the probability of discordance. For example, the probability of discordance can even be less than 1% in specific subgroups of patients.

Based on the results of the logistic regression model and the low percentages of patients with discordant test results that were found in this study, performing multiple tests is probably unnecessary for the majority of patients. However, a number of significant risk factors for discordance were identified in this study, suggesting that testing twice is likely beneficial in very specific subgroups of patients. As a next step, discordance risks should be validated in future studies, focusing particularly on patients with high predicted discordance risks, to further tailor multiple ER/PR/HER2 testing.

Potential cost savings of omitting tests can potentially sum up to approximately €205,000 yearly. However, prices used in this calculation are list prices, which are not always reflecting the actual money paid for each test. In addition, potential cost savings were based on the assumption that discordance can be perfectly predicted, whereas it is hard to make perfect predictions in practice. Furthermore, to be able to estimate full potential cost impact, treatment decisions based on discordance in these test results and guideline adherence regarding the use of these tests should also be taken into account.

No adjuvant treatment decisions based on the discordance in this study were taken into account. Future studies should focus on the impact of discordant results on future treatment decisions. In addition, no survival results could yet be derived for this cohort of patients as these patients were diagnosed after January 12, 2016. Consequently, the follow-up period of this patient cohort is (yet) too short to draw firm conclusions regarding survival. Future studies should incorporate the impact on treatment decisions and associated survival outcomes in further predicting the probability of potential discordance. In addition, further treatment decisions related to discordance should be taken into account. Especially when treatment decisions will not differ, even though test results are discordant, additional testing may be omitted.

## Conclusions

Testing for ER/PR/HER2 on both tumor biopsy and tumor resection material is not performed in the majority of patients. The discordance in test results of the ER/PR/HER2 status in patients with invasive breast cancer is limited. Of all three receptors, remarkably the PR receptor status is most frequently measured on both biopsy and tumor resection material and was subsequently found to be discordant most frequently. In addition, the probability of a discordant test result is generally low, but can, however, be significantly influenced by several patient and tumor characteristics.

Future research investigating either discordance, their corresponding predictive models, or its potential consequences should also take further treatment decisions and treatment outcomes into account for each of the receptors.

## Data Availability

The datasets used and analyzed in this study are not publicly available but can be requested at PALGA.

## Abbreviations

ASCO: American Society of Clinical Oncology
CAP: College of American Pathologists
ER: Estrogen
FISH: Fluorescent in situ hybridization
HER2: Human epidermal growth factor 2
IHC: Immunohistochemistry
ISH: In situ hybridization
PALGA: Dutch pathology registry
PR: Progesterone
